# Gallium-68 DOTATATE Production with Automated PET Radiopharmaceutical Synthesis System: A Three Year Experience

**Published:** 2014

**Authors:** Alireza Aslani, Graeme M Snowdon, Dale L Bailey, Geoffrey P Schembri, Elizabeth A Bailey, Paul J Roach

**Affiliations:** 1 Department of Nuclear Medicine, Royal North Shore Hospital, Sydney, Australia; 2 Sydney Medical School, University of Sydney, Sydney, Australia; 3 Faculty of Health Sciences, University of Sydney, Sydney, Australia

**Keywords:** DOTATATE, Automated synthesis systems, Gallium-68, Neuro-endocrine tumours, Pet radiopharmaceuticals

## Abstract

**Objective(s):**

Gallium-68 (Ga-68) is an ideal research and hospital-based PET radioisotope. Currently, the main form of Ga-68 radiopharmaceutical that is being synthesised in-house is Ga-68 conjugated with DOTA based derivatives. The development of automated synthesis systems has increased the reliability, reproducibility and safety of radiopharmaceutical productions. Here we report on our three year, 500 syntheses experience with an automated system for Ga-68 DOTATATE.

**Methods:**

The automated synthesis system we use is divided into three parts of a) servomotor modules, b) single use sterile synthesis cassettes and, c) a computerised system that runs the modules. An audit trail is produced by the system as a requirement for GMP production. The required reagents and chemicals are made in-. The Germanium breakthrough is determined on a weekly basis. Production yields for each synthesis are calculated to monitor the performance and efficiency of the synthesis. The quality of the final product is assessed after each synthesis by ITLC-SG and HPLC methods.

**Results:**

A total of 500 Ga-68 DOTATATE syntheses (>800 patient doses) were performed between March 2011 and February 2014. The average generator yield was 81.3±0.2% for 2011, 76.7±0.4% for 2012 and 75.0±0.3% for 2013. Ga-68 DOTATATE yields for 2011, 2012, and 2013 were 81.8±0.4%, 82.2±0.4% and 87.9±0.4%, respectively. These exceed the manufacturer's expected value of approximately 70%. Germanium breakthrough averaged 8.6×10^-6^% of total activity which is well below the recommended level of 0.001%. The average ITLC-measured radiochemical purity was above 98.5% and the average HPLC-measured radiochemical purity was above 99.5%. Although there were some system failures during synthesis, there were only eight occasions where the patient scans needed to be rescheduled.

**Conclusion:**

In our experience the automated synthesis system performs reliably with a relatively low incident of failures. Our system had a consistent and reliable Ga-68 DOTATATE output with high labelling efficiency and purity. There is minimal operator intervention and radiation exposure. The system is GMP-compliant and has low maintenance and acceptable running costs. This system together with the recommended ^68^Ge/^68^Ga generator is well suited for use in a hospital-based radiopharmacy.

## Introduction

Today, the majority of Positron Emission Tomography (PET) studies are performed with Fluorine-18 (F-18) labelled radiophar-maceuticals and, in particular, fluoro-deoxyglucose (FDG). F-18 is a cyclotron-produced radioisotope and, as a result, it requires an on-site cyclotron or access to a nearby cyclotron and subsequent delivery, costing both time and money. Generator-produced radioisotopes, such as Ga-68, have a number of advantages that makes them attractive as hospital-based PET radioisotopes.

Ga-68 has rapidly gained attention as one of the ideal research and hospital-based PET radioisotopes. It has the advantage of being produced on-site from a small wet-bed ^68^Ge/^68^Ga generator, so it can be made available within minutes as well as at much lower cost than other similar radioisotopes. It has a short half-life (67.7 minutes) making it suitable for human studies and low radiation dose to the patients. The trivalent nature of Gallium-68 also makes it well suited for radiolabelling the 1,4,7,10-tetraazacyclododecane-1,4,7,10-tetra acetic acid (DOTA) compounds as well as labelling proteins and peptides (1).

**Figure 1:**
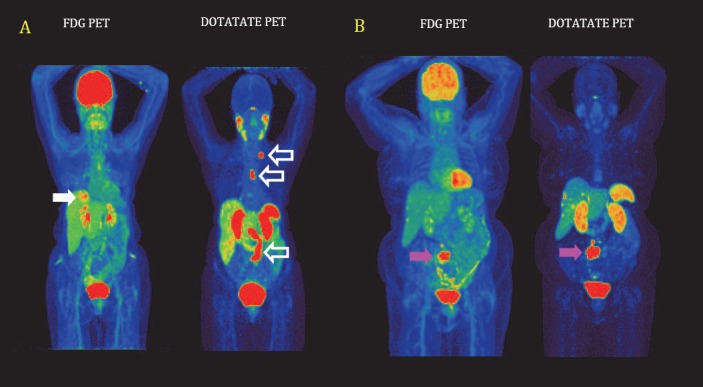
The images show two patients who underwent contemporaneous [F-18]-FDG and [Ga-68]-DOTATATE imaging. (A) This is a 72 year old female patient diagnosed with carcinoid and known metastatic disease in the liver and abdominal lymph nodes. The liver lesion (closed white arrow) was identified on FDG imaging alone. Lymph nodes (open white arrows) with increased focal uptake were only demonstrated on DOTATATE imaging. FDG positive / DOTATATE negative lesions are more likely to be poorly differentiated and higher grade whereas DOTATATE positive / FDG negative are usually well differentiated and lower grade. (B) A 73 year old female patient with metastatic small bowel carcinoid, currently being treated with [Lu-177]-DOTATATE therapy. The known small bowel lesion can be seen on both the FDG and DOTATATE images (pink arrows). Used in combination, FDG and DOTATATE imaging allows the degree of differentiation of the lesions to be assessed which is a guide to the most appropriate form of therapy (Peptide Receptor Radionuclide Therapy (PRRT) or conventional chemotherapy). All patient scans were performed in the Department of Nuclear Medicine, Royal North Shore Hospital, Sydney using a Siemens Biograph mCT Time-of-Flight PET scanner (*Biograph mCT.s/x 64, Siemens Healthcare, IL, USA*)

Currently a general review of the reports published in the literature suggests that the main non-cyclotron produced PET radiophar-maceutical that is being synthesised in-house is Ga-68 conjugated with DOTA based derivatives, specifically for imaging over-expression of various subtypes of somatostatin receptors expressed by neuroendocrine tumours (NETs). The main radiopharmaceuticals being used for this application are DOTATATE, DOTATOC and DOTANOC, all of which are labelled via the DOTA chelator to Ga-68. Figure 1 shows examples of patient scans from our institution using both FDG and DOTATATE, demonstrating that these tumours can be either FDG or DOTATATE positive or negative in various combinations. Although the synthesis and purification of these radiopharmaceuticals can successfully be carried out manually in the laboratory, the use of automated synthesis systems (2), (3) is on the increase. These automated systems have clear advantages over the manual methods which have resulted in their increasing installation and use in hospital-based radiopharmacies. Here we report on our three year experience of over 500 syntheses with an automated synthesis system for Ga-68 DOTATATE.

## Methods

There are a number of reports in the literature using and detailing various automated synthesis systems. One of the automated systems that closely, but not exactly, resembles our system was reported by Decristoforo et al (4). Automated synthesis systems are generally set-up and adjusted to the specific needs of the institution. As a result over time, these setups evolve and are tweaked to improve productivity and become unique to the institution. The system used here was the Eckert & Ziegler Eurotope's Modular-Lab Pharm Tracer® automated synthesis system.

All materials and methods used were followed as instructed or later modified by Eckert & Ziegler Eurotope (Berlin, Germany) from whom the automated synthesis system and synthesis cassettes were obtained.

### Reagents

As recommended by the manufacturer of the automated synthesis system, Eckert & Ziegler Eurotope (Berlin, Germany), all reagents were high purity pharmaceutical grade, unless stated otherwise. The exact grade as well as the source of the reagents used was their recommendation.

Water puriss p.a (FLUKA Trace SELECT® 95305) used in the preparation of the majority of reagents was obtained from Sigma-Aldrich, Australia. Hydrochloric acid (Ultrapure 30% HCl, 0.1 M), was obtained from Merck kGaA (64271 Darmstadt, Germany). In addition, the >99.5% pure acetone (puriss p.a 32201), 200 proof ethanol (HPLC / spectrophotometric grade, 459828-1L), glacial acetic acid solution (>99.99% purity; 338826) and sodium acetate trihydrate (BioUltra® >99.5% purity; 71188) were obtained from Sigma-Aldrich, Australia. Sodium chloride 0.9% for injection in 50mL glass bottles (INJ089) were obtained from Phebra (Lane Cove, NSW, Australia).

The reagents used for the High Pressure Liquid Chromatography (HPLC) phase of the automated system were of HPLC grade. The trifluoroacetic acid (HPLC grade FLUKA; 91707), acetonitrile (CHROMASOLV®, HPLC grade, >99.9% purity; 34998), ammonium acetate (17836), and HPLC grade water (CHROMASOLV®; 270733) were obtained from Sigma-Aldrich, Australia. The sodium citrate dihydrate p.a was obtained from Bacto Laboratories (Liverpool, NSW, Australia; #1.06448)

The DOTATATE, or DOTA-(Tyr^3^)-octreotate (where DOTA=1, 4, 7, 10 - tetraazacyclo-dodecane – 1, 4, 7, 10-tetraacetic acid) was obtained from Auspep, Victoria, Australia. The peptide is obtained as a 1 mg powder which is subsequently dissolved in 1 mL of water and used in 40 µl aliquots. Up till recently the DOTATATE was only available as a research grade peptide. However, since mid-2013 a Good Manufacturing Process (GMP) grade product has also been available which we now utilise.

The preparation of the required Ga-68 DOTATATE reagents and chemicals are shown in Table 1. This table is used as a quick instruction guide for the daily preparation of chemicals at our centre.

**Table 1:** Preparation methods for Ga-68 DOTATATE chemicals

Name of Solution	Preparation	Product Code
**HCl (0.1M)**	1.06 mL 30% HCl	**30%, HCl Ultra pure, Merck 1.01514.0500**
	Fill up to 100 mL with H_2_O	**Water Puriss. p.a., Sigma Aldrich FLUKA 95305**
**Eluent (0.02M HCl, 98% acetone)**	52.5 μL HCl	**30%, HCl Ultra pure, Merck 1.01514.0500**
	557 µL H_2_O	**Water Puriss. p.a., Sigma Aldrich FLUKA 95305**
	Volumetrically fill up to 25 mL with acetone ***Store in freezer. Replace each Monday morning Do not use beyond 5 days.***	**99.5% Acetone, puriss. p.a., Sigma Aldrich 32201**
**Buffer stock solution** **(Sodium acetate buffer 0.2M)**	**Prepare 2 solutions**	
	**A.** 0.2 M NaOAc:	**Sodium acetate trihydrate, Sigma Aldrich 71188 [250g] Puriss. p.a., Sigma Aldrich FLUKA 95305** **Acetic acid solution, glacial 99.99+%, Sigma Aldrich 338826 100mL** **Water Puriss. p.a., Sigma Aldrich FLUKA 95305**
	2.721 g NaOAcx3H_2_O	
	Fill up to 100 mL with H_2_O	
	**B.** 0.2 M AcOH:	
	1.145 mL AcOH	
	Fill up to 100 mL with H_2_O	
	**Mix the 2 solutions:**	
	82mL 0.2 M AcOH +	
	18mL 0.2 M NaOAc	
	Check the pH=4.0 ± 0.2. **If pH is not in range do not use the solution. Store in fridge.**	
**Ethanol solution**	10 mL ethanol	**Ethanol 200 proof HPLC/Spectrophotometric grade Sigma Aldrich 459828-1L**
	10 mL H_2_O	**Water Puriss. p.a., Sigma Aldrich FLUKA 95305**
**NaCl (saline)**	50 mL sterile saline	**Sodium Chloride 0.9% Injection 900mg in 50mL Phebra INJ072**
**DOTATATE**	Stock DOTATATE 1 mg peptide in 1 mL H_2_O. ***Store in freezer***	**DOTA-(TYR3) Octreotate acetate salt 2500437 1mg CAS-No 177943-89-4**
	Dispense 40 μL into eppendorf vials. ***Store in freezer***	**Water Puriss. p.a., Sigma Aldrich FLUKA 95305**
**HPLC For ^68^Ga-DOTATATE** **22% Acetonitrile**	Fill 385 mL of water in a 1000 mL cylinder.	**WATER, CHROMASOLV, FOR HPLC Sigma Aldrich # 270733-4L**
	Add 0.5 mL TFA by pipette	**TFA (trifluoroacetic acid, HPLC Grade, Sigma Aldrich FLUKA, 91707)**
	Fill to 500 mL with acetonitrile	**Acetonitrile (HPLC Grade, Chromosolv R Plus Sigma Aldrich #34998)**
**Preparation of HPLC column cleaning solution**	Fill **700 mL** of acetonitrile in a 1000 mL cylinder.	**Acetonitrile (HPLC Grade, ROTH, 8825.2)**
	Fill to **1000 mL** with water	**WATER, CHROMASOLV, FOR HPLC Sigma Aldrich # 270733-4L**
**68Ga-Dotatate ITLC QC**	**0.1 M Na Citrate pH=5.0 (2.94 g in 100 mL)**	**Sodium citrate dehydrate p.a Bacto Laboratories #1.06448**
	1 M ammonium acetate in water / methanol, 1:1 * 1 M ammonium acetate 7.704g in 100 mL water	**Ammonium Acetate Sigma Aldrich #17836**

### Synthesis Cassettes and Automation

The synthesis of DOTATATE is performed using the Eckert & Ziegler Eurotope's Modular-Lab Pharm Tracer® automated synthesis system designed specifically for the synthesis of radiopharmaceuticals. It can be separated into three parts. These are: a) the modules assembled according to the specific radiopharmaceutical being synthesised; b) the synthesis cassettes on which the appropriate chemicals, reagents, filters, columns, etc are attached; and c) the computerised system that runs the modules which in turn run the valves and syringes on the cassettes producing the required radiopharmaceutical. Since the system is computer-controlled, an audit trail is always maintained which is a requirement for GMP production runs.

There is a specific synthesis cassette for the production of Ga-68 DOTATATE as well as one for a simple elution of the Ga-68 generator. The system is also currently being used for the production of other radiopharmaceuticals, such as Lu-177 DOTATATE, using a specific synthesis cassette. The production cassettes are obtained from Eckert & Ziegler Eurotope. These cassettes are sterile and single-use.

As with the cassettes, there is a specific template computer program for each specific radiopharmaceutical synthesis process. These synthesis templates are also provided by Eckert & Ziegler Eurotope. These are subdivided into template programs for the cassette's pressure testing, terminal sterilisation filter testing, running the HPLC analysis and eluting the generator. The Modular-Lab software also allows programming and modifications to the templates via a graphical user interface (GUI).

### Generators

So far, on average one generator per year has been used during the three years of using the automated system. All three ^68^Ge/^68^Ga generators were obtained from Eckert & Ziegler Eurotope (Berlin, Germany). The first generator (IGG100-30M) used was a 1.11 GBq whereas the subsequent two (IGG100-50M) generators were 1.85 GBq. The latter two generators allowed for larger number of patient doses per synthesis and a longer “useful” life.

Due to the build-up of metal ions on the column, the generators are required to be eluted on a daily basis and within 24 hours prior to any DOTATATE synthesis. Therefore, the generator was eluted on the first day of the week and on non-synthesis days. The elution process involved using an elution cassette, 0.1 M HCl solution driven by the Modular-Lab system in approximately five minutes. The elution volume was 8 mL.

### Automated Radiolabelling Process

The radiolabelling process is explained fully and in details in the instruction manual that is supplied with the Modular Lab Pharm Tracer® automated system. Briefly the process is summarised here.

**Figure 2:**
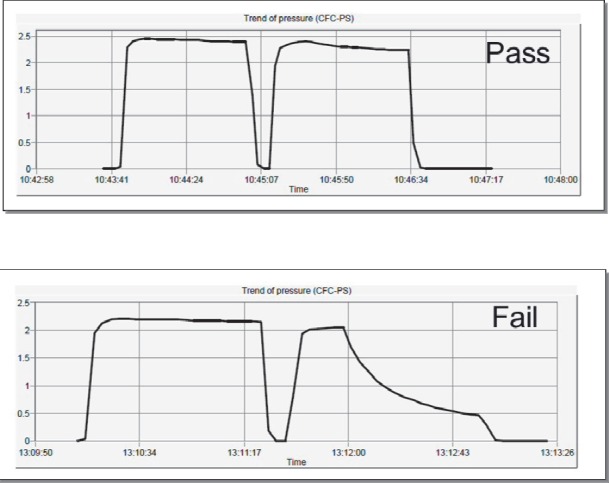
Pressure test trace indicating a pressure test Pass and a Fail

Once the sterile synthesis cassette is removed from its packaging and all connections tightened, it is attached to the Modular Lab's synthesis cassette module. The radiolabelling process is performed in three steps. The first step tests the cassette for any leaks. This step, also known as the Cassette Pressure Test, applies a pressure of 200 kPa to various sections of the cassette. This test was performed by attaching the cassette to a high purity nitrogen gas supply which its pressure regulator has been adjusted to deliver 200 kPa of pressure. The Modular Lab Pharm Tracer®'s software then drove the pressure and synthesis modules to pressure test the cassette. A visual progress graph was also displayed. If there was no loss of pressure below 100 kPa, the test was considered to have passed and cassette was suitable for synthesis (Figure 2). However, if the test fails, the cassette is removed from the module, all connections re-tightened and test repeated. If the test fails again, the cassette was rejected and sent back to the manufacturer and replaced. On passing the test, the necessary pre-prepared reagents, 0.9% saline, ethanol, eluent, and DOTATATE peptide in acetate buffer are placed on the appropriate sections of the synthesis cassette and ^68^Ge/^68^Ga generator tube connected. The empty containers, the waste bottle and product vial are then attached. The process of pressure testing the cassette takes approximately five minutes. On completion, the cassette pressure test process log is saved on the computer for audit trail purposes.

**Figure 3:**
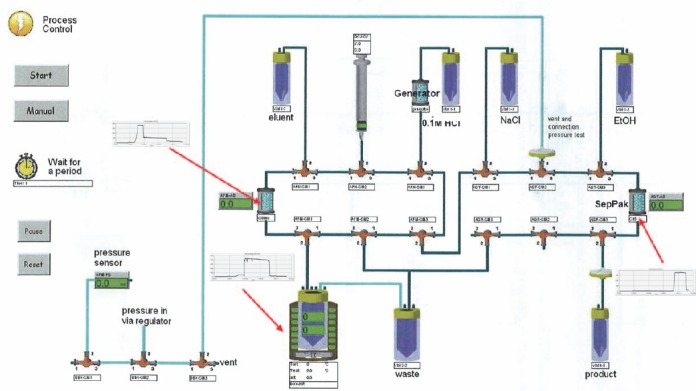
Modular Lab Pharm Tracer® Ga-68 DOTATATE synthesis schematic. Imaged captured from Eckert & Ziegler Eurotope's Modular-Lab Pharm Tracer® automated synthesis system and used with permission

**Figure 4:**
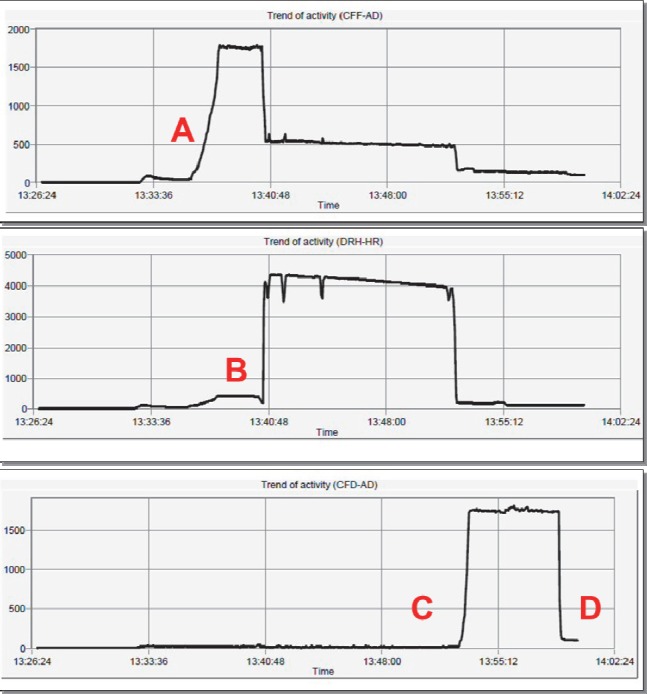
A: Concentration of ^68^GaCl_3_ from generator onto Strata X C cation exchange column; B: Elution of ^68^GaCl_3_ with acetone: HCl eluent into reaction vial containing DOTA peptide in buffer and 0.2 mL ethanol; C: Transfer of Ga-68 DOTATATE to SepPak® cartridge; D: Elution of SepPak® to the product vial with ethanol followed by saline

The second step of the synthesis phase is also driven by the Modular Lab Pharm Tracer®software. The duration of this process is 33 minutes and is the main step of the entire process. The movement of all liquids is driven by a 10 mL syringe attached to the syringe-driver module. This phase involves a number of different steps, including washing of various sections with 0.9% saline. Briefly, the Waters tC18 Light reverse phase silica cartridge (also known as C18 ion exchange or SepPak® cartridge) is initially primed (wetted) with ethanol followed by 0.9% saline. The generator is then eluted with 8 mL of 0.1 M HCl and passed through the Strata X C ion exchange cartridge trapping the gallium-68 chloride. The excess 0.1 M HCl is then passed to the waste bottle. The gallium-68 chloride is released from the cartridge with 0.4 mL of the eluent (acetone/HCL) and into the reaction vial containing the DOTATATE peptide in acetate buffer. The mixture is then heated for 400 seconds at 95^º^C to bind the DOTATATE with gallium-68 chloride and to evaporate the eluent (acetone/HCL). The contents of the reaction vial are then removed and passed through the C18 ion exchange cartridge where the Ga-68 DOTATATE is trapped and the unbound Ga-68 and DOTATATE are sent to the waste bottle. The trapped Ga-68 DOTATATE is eluted from the C18 ion exchange by ethanol and then 0.9% saline into the product vial via a 0.22 µm filter. The total volume of the final product is 8 mL. The Modular Lab Pharm Tracer® software provided a graphical display of each step and progress of the synthesis (Figure 3). On completion, the synthesis process is saved on the computer for audit trail purposes (Figure 4).

The third phase of the synthesis involves testing of the integrity of the 0.22 µm filter. This is known as the Filter Pressure Test and is, again, driven by the Modular Lab Pharm Tracer® software. The only operator intervention required is the removal of the 0.22 µm filter and needle from the product vial and putting it into the waste bottle prior to the test. The test is performed by subjecting the filter to 200 kPa of pressure. If pressure is maintained above 100 kPa, the test is considered to have been successful and the product is sterile for patient use. However, if the pressure falls below 100 kPa, the test is considered to have been unsuccessful and there is a chance of the product being unsterile. To rectify this, the product needs to be drawn-up manually in a 10 mL syringe and passed through a new 0.22 µm filter into another sterile vial. To date, this has never occurred at our centre. On completion, the filter pressure test process log is saved on the computer for audit trail purposes. The trace produced by the software is identical to the ones produced for the Cassette Pressure Test (Figure 2). After removing a 0.2 mL sample from the product for quality control purposes, the product is ready to be administered to the patient.

### Analysis and Quality Control

The quality control of the resulting product is done by two methods of Instant Thin Layer Chromatography (ITLC) as well as High Pressure Liquid Chromatography (HPLC). These are performed using the 0.2 mL sample taken directly from the final Ga-68 DOTATATE product.

### Instant Thin Layer Chromatography (ITLC)

The ITLC test is used to determine the percentage of Ga-68 DOTATATE, Ga-68 impurities, and Ga-68 colloid in the final product. The ITLC paper strips are counted in a laboratory gamma-counter (PerkinElmer Wizard^2^® Automated Gamma Counter, PerkinElmer Downers Grove, IL USA) and results entered into a spread sheet (Microsoft Excel Version 2010) for final calculation and analysis.

For the determination of percentage Ga-68 DOTATATE and Ga-68 impurities, 0.1 mol/L citrate buffer (pH=5) with ITLC-SG paper (Pall Corporation, East Hills, New York) is used; free Ga-68 (R_f_=0.8–1), Ga-68 peptide (R_f_=0.0–0.3) and Ga-68 colloid (R_f_= 0.0-0.2).

For the determination of percentage Ga-68 colloid, 1 mol/L ammonium acetate / methanol (1 : 1) / ITLC-SG (Pall Corporation) is used; Ga-68 colloid (R_f_= 0.0-0.2), Ga-68 peptide (R_f_= 0.8-1) and free Ga-68 (R_f_=0.8–1).

### High Pressure Liquid Chromatography (HPLC)

The HPLC module was provided by Eckert & Ziegler Eurotope and was driven by the Modula Lab Pharm Tracer® software. This was a Knaur Smartline D14163 pump (Knaur, Berlin, Germany) with Knaur Online Degasser V7620 (Knaur, Berlin, Germany), ACE3 C18 column 150×3 mm (Advanced Chromatography Technology, Aberdeen, UK) flow rate 0.6 mL min^-1^, water / acetonitrile (ACN) / 0.1% trifluoroacetic acid with 22% ACN; 0.04 mL sample is used to determine the percentage Ga-68 DOTATATE content of the final product.

### Ge-68 Breakthrough

The Ge-68 breakthrough is measured in an eluted sample, on a weekly basis, after a complete Ga-68 decay (>48 hours). The measurements are carried out in a laboratory gamma-counter (PerkinElmer Wizard^2^® Automated Gamma Counter, PerkinElmer Downers Grove, IL USA) and the data entered into and calculations made using a Microsoft Excel version 2010 spread sheet.

## Results

A total of approximately 500 Ga-68 DOTATATE syntheses were performed between March 2011 and April 2014 in our laboratory. All syntheses were carried out using the Eckert & Ziegler Eurotope's Modular Lab Pharm Tracer® automated synthesis system and software. All syntheses were performed in the Department of Nuclear Medicine, Royal North Shore Hospital in a purpose-built radiopharmaceutical laboratory.

### Generators

**Figure 5:**
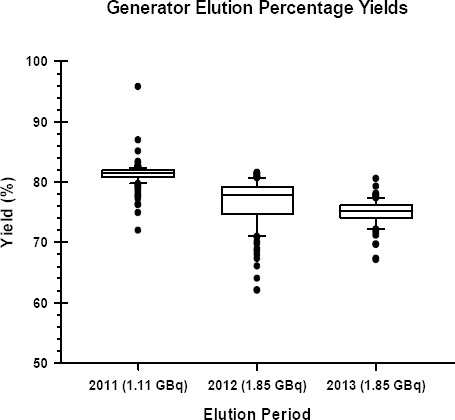
Box and whiskers plot of the percentage yield for each generator. The generators for the second and third years were identical

Generator yields for the three years are shown in Figure 5. The percentage yield was calculated as the actual yield as a percentage of calculated as the actual yield as a percentage of the expected yield. The average generator yield for the first year was 81.3±0.2%. The yields were lower for second and third years at 76.7±0.4% and 75.0±0.3% corresponding to the larger generators. The expected yield, as indica-ted by the manufacturer, is approximately 70%.

### Ga-68 DOTATATE Syntheses

Although the synthesis cassettes used for Ga-68 DOTATATE had the same overall design and specifications there were some minor modifications made by the manufacturer during the three years. These modifications were made as a part of ongoing improvements and quality control in response to user comments and feedback. Some of these modifications addressed the connections on the cassettes to increase reliability whereas some addressed the increase in product yield by, for example, increasing the volume of ethanol being used to elute the final product from the C18 cartridge. These resulted in the cassette failure rates dropping dramatically with time and are now very rare. In addition, these also resulted in the average synthesis yields increasing from 81.8 ± 0.4% in the first year to 82.2±0.4% (*P*=0.42) in the second year and 87.9±0.4% (*P*<0.001) in the third year (Figure 5).

**Figure 6:**
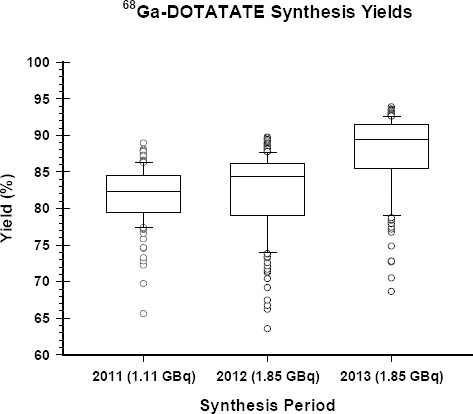
Ga-68 DOTATATE synthesis yields for the automated synthesis system

Due to the nature of the ^68^Ge/^68^Ga generator and the half-life of 271 days when new, enough activity can be obtained to synthesise sufficient Ga-68 DOTATATE for three patients per synthesis run (150 MBq to 200 MBq per dose). As the generator decays this is reduced to two patients after approximately one year and eventually to one patient. This reduces the cost- effectiveness of the synthesis process requiring the purchase of a new generator. In order to predict how long the generator will be effective in producing enough Ga-68, a graph of final product activity against the age of generator (Figure 6) was made and an equation of the line of best-fit was obtained. This took the average synthesis yields into account and produced a rough estimate as to when a new generator would be required to maintain the minimum required number of patients per synthesis run. For example, with a 1.85 GBq generator a replacement is required at approximately 14 months to provide enough product for more than one patient per run.

### Germanium-68 Breakthrough

The germanium-68 breakthrough measured during the three years of use was constantly well below the recommended level of 0.001% of the total radioactivity. The actual average values were 8.6×10^-6^ % of the total radioactivity.

### Radiochemical Quality Control

**Figure 7:**
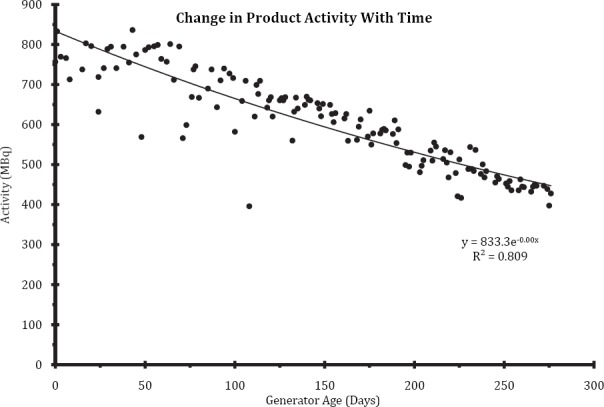
Graph to predict the activity Ga-68 DOTATATE synthesis product with time (1.85 GBq^68^Ge/^68^Ga generator)

The radiochemical purity of the final product was measured using ITLC as well as HPLC. The results demonstrated (Figure 7) that the average radiochemical purity as determined by ITLC was above 99% for 2011 (99.6±0.2%) and 2012 (99.1±0.2%) and above 98% for 2013 (98.7±0.4%). The average radiochemical purity as determined by HPLC technique was above 99.5% for all three years (Figure 9). The values for 2011 and 2012 were 99.75±0.03% and 99.71±0.04%, respectively. The 2013 period's HPLC results were marginally higher at 99.87±0.01%.

**Figure 8:**
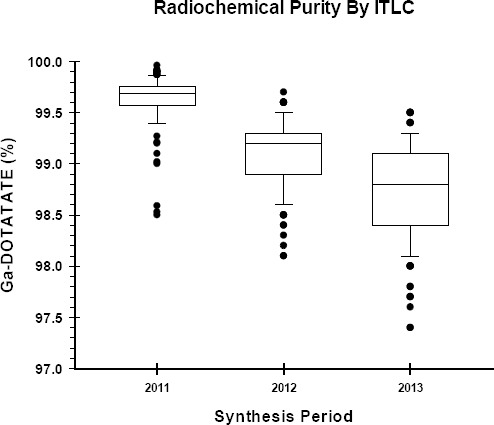
ITLC test to determine radiochemical purity of Ga-68 DOTATATE

**Figure 9:**
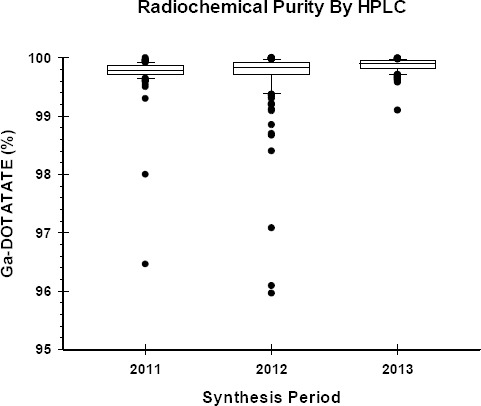
HPLC test to determine radiochemical purity of Ga-68 DOTATATE

These results were well above the minimum recommended value of 91% (5). The reason for the small decline in the ITLC-measured purity level during the three years has not been identified.

### Cassette and System Failures

The initial teething problems were mainly associated with cassette leaks. There were leaks from the cassette on three occasions during synthesis. With operator intervention, the leaks were rectified mid-synthesis resulting only in a minor loss of final product. The cassette pressure test step of the synthesis was designed to stop such leaks and performed successfully in the vast majority of cases.

From the 500 syntheses performed, only eight failures (1.6%) led to patient scans being deferred. From these, there were two due to leaks at the C18 cartridge connection, one leakage at the Strata X C connection, one leakage at the T-connection to the waste bottle, one due to the eluent not being drawn into the Strata X C cartridge, one due to all Ga-68 ending-up in the waste bottle (cause unidentified), one due to the syringe driving spontaneously halting, and one high activity retention at the C18 cartridge.

**Figure 10:**
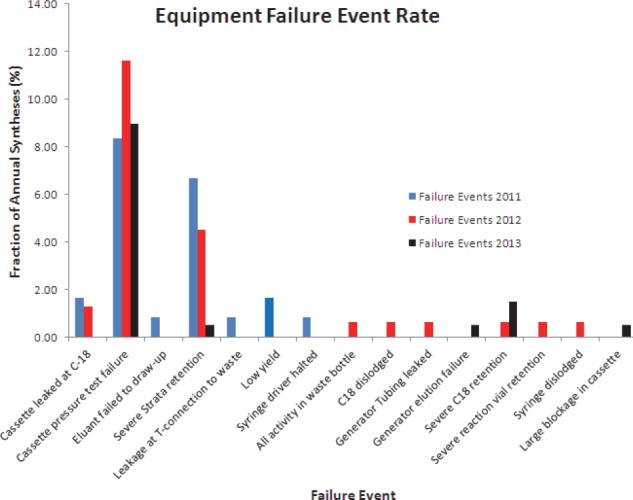
Rate and type of failures during the three synthesis years. These failures were due to either cassettes or Modular Lab System not performing as required and did not lead to patient deferment

There were different types of failures associated with the Modular Lab System and the synthesis cassette. The majority were cassette-related failures (Figure 10). The predominant failure with the cassettes over all three years was the cassette pressure test failure. The only two events that were associated with the Modular Lab System was first the syringe drive spontaneously halting and second, the syringe spontaneously falling off the syringe drive during the synthesis process. The latter was rectified by operator intervention whereas the former could not and led to total system failure.

### Record Keeping and Audit Trail

As a part of a GMP system a good record keeping and an audit trail is mandatory. For this purpose electronic copies, in Adobe PDF format, of the pressure tests, syntheses, and HPLC tests were automatically recorded by the automated synthesis system. In addition, patients’ details, quality control results (ITLC and HPLC), system failures, and synthesis and pressure test batch numbers were recorded in a laboratory log book. This was predominantly for fast access to previous work. A summary of the above was also recorded on a spread sheet (Microsoft Excel Version 2010) and electronically saved on the synthesis PC as well as a hardcopy printed and stored at the Department of Nuclear Medicine.

## Discussion

Gallium-68 has some significant benefits over other diagnostic radioisotopes, especially those used for PET scanning. Due to its short half-life all radiolabelling of Ga-68 radiopharmaceuticals is required to be performed in a short period of time. The ^68^Ge/^68^Ga generator has an approximately seven hour regeneration time. This means that once it is eluted for synthesis, the next elution or synthesis can only be made after seven hours for a full elute. Therefore, the synthesis cannot be repeated (at least not without compromising yield) if there are any errors or failures. The automated synthesis systems are likely to be more reliable and reproducible and can ensure consistent syntheses yields.

**Figure 11:**
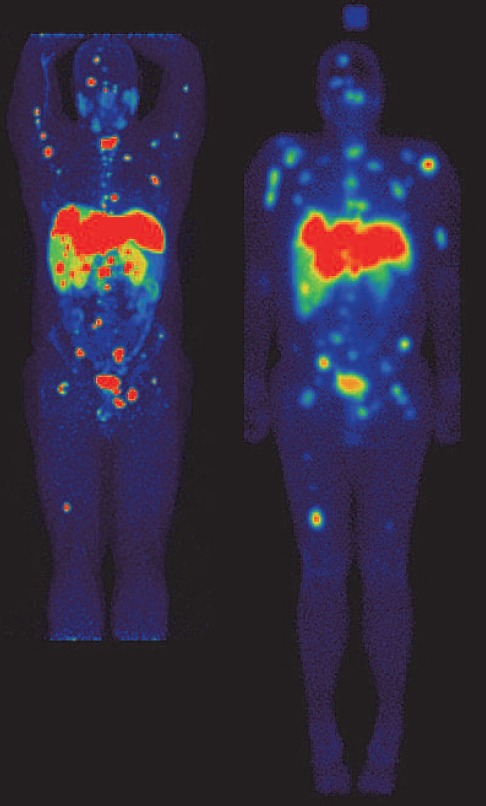
Ga-68 (left) and Lu-177 (right) DOTATATE images, in the same patient, are shown. The Ga-68 image was obtained with a PET scanner and a Maximum Intensity Projection (MIP) image from the anterior orientation is shown. The Lu-177 image is a geometric mean of anterior and posterior whole body planar (2D) scans acquired approximately four hours after the infusion of 8 GBq of DOTATATE ceased. The estimate of the retained radioactivity at the time of scanning is ~6 GBq. Apart from the obvious differences in spatial resolution between the two devices the biodistribution is similar. The rectangular area above the head on the Lu-177 gamma camera image is a calibration standard

The automated synthesis systems have the additional benefit that they can be readily adapted to be used for the synthesis of other radiopharmaceuticals (6). Lu-177 DOTATATE is one example. We have recently commenced synthesising Lu-177 DOTATATE using the same automated synthesis system as the Ga-68 DOTATATE using a Lu-177 DOTATATE-specific synthesis cassette. The Lu-177 DOTATATE is used for neuroendocrine tumour (NET) therapy. Due to the presence of a gamma-ray component from Lu-177, the patient can be imaged using a gamma camera. This is useful for monitoring the patient's progress. This is demonstrated in Figure 11 where the patient has been diagnosed using a Ga-68 DOTATATE and subsequently has received Lu-177 DOTATATE therapy. These images demonstrate the theranostic imaging potential of diagnostic Ga-68 (PET) and therapeutic Lu-177 (gamma camera) DOTATATE. The Lu-177 image shown was acquired approximately four hours after the DOTATATE infusion ceased. Allowing for differences in the spatial resolution between the PET camera and gamma camera, the distributions are strikingly similar.

Although there were a number of production failures, almost all were associated with the synthesis cassettes and were recoverable. The majority of these failures were detected before the commencement of the syntheses. These were rectified either by minor adjustments to the cassettes or the cassettes were replaced. Failures resulting in patient cancellation were extremely rare.

All three generators used were obtained from the same manufacturer. The average yields for all three were above the manufacturer's quoted estimate of 70%. The germanium breakthrough for all there generators were within the required specifications of less than 0.01%. The highest breakthrough level that measured in this period was 0.0007%.

The synthesis yields were similar for the first two years but increased in the third year. This was achieved by the manufacturer modifying the synthesis software to pass a slightly higher volume of ethanol through the C18 cartridge. The net effect of this was that more of the product concentrated on the cartridge will be released thus increasing the product yield. The manufacturer also made slight modifications to the synthesis cassettes. These modifications mainly involved how the tubes are connected to the cassettes. These reduced the rate of leaks and cartridges dislodging.

The actual Modular Lab System, with the exception of two occasions, performed flawlessly throughout the three years without any maintenance. However, the synthesis cassettes had relatively much higher failure rates. As a percentage of total number of syntheses carried out, these failure rates were very low (16.4%). From the total of 82 equipment failure events in the three year period, only eight (9.8%) led to a full synthesis failure and patients being deferred. This rate is also very low and all occurred in initial teething period. With more operator experience during the second and third years, some of the failures that could have led to full synthesis failures were successfully averted. In addition, feedback to the manufacturer led to a number of areas of concern being addressed – particularly those relating to the cassettes.

The Ga-68 DOTATATE product radiochemical purity was measured using the ITLC as well as the HPLC techniques. Results showed that not only was the synthesised product above the minimum recommended level, but also was of high purity. There was, however, a marginal and gradual decline in ITLC values. We could not explain this decline. The very low variation in the product quality (Figure 8, 9) demonstrated a consistent-quality production system that could reliably produce a very high quality product.

## Conclusion

The automated synthesis system provides a reliable and straightforward approach to synthesising one particular short-lived PET radiopharmaceutical. Eckert & Ziegler Eurotope's Modular-Lab Pharm Tracer® is an innovative automated synthesis system that performs reliably with a relatively low incident of failures. Our system had a consistent and reliable Ga-68 DOTATATE output with high purity exceeding the minimum recommended requirements. The system is GMP-compliant and has low maintenance and acceptable running costs.
